# Quantifying the mosquito’s sweet tooth: modelling the effectiveness of attractive toxic sugar baits (ATSB) for malaria vector control

**DOI:** 10.1186/1475-2875-12-291

**Published:** 2013-08-23

**Authors:** John M Marshall, Michael T White, Azra C Ghani, Yosef Schlein, Gunter C Muller, John C Beier

**Affiliations:** 1 Department of Infectious Disease Epidemiology, MRC Centre for Outbreak Analysis and Modelling, Imperial College London, London, UK; 2 Department of Parasitology, Kuvin Center for the Study of Tropical and Infectious Diseases, Hadassah Medical School, Hebrew University, Jerusalem, Israel; 3 Department of Epidemiology and Public Health, Miller School of Medicine, University of Miami, Miami, Florida, USA

## Abstract

**Background:**

Current vector control strategies focus largely on indoor measures, such as long-lasting insecticide treated nets (LLINs) and indoor residual spraying (IRS); however mosquitoes frequently feed on sugar sources outdoors, inviting the possibility of novel control strategies. Attractive toxic sugar baits (ATSB), either sprayed on vegetation or provided in outdoor bait stations, have been shown to significantly reduce mosquito densities in these settings.

**Methods:**

Simple models of mosquito sugar-feeding behaviour were fitted to data from an ATSB field trial in Mali and used to estimate sugar-feeding rates and the potential of ATSB to control mosquito populations. The model and fitted parameters were then incorporated into a larger integrated vector management (IVM) model to assess the potential contribution of ATSB to future IVM programmes.

**Results:**

In the Mali experimental setting, the model suggests that about half of female mosquitoes fed on ATSB solution per day, dying within several hours of ingesting the toxin. Using a model incorporating the number of gonotrophic cycles completed by female mosquitoes, a higher sugar-feeding rate was estimated for younger mosquitoes than for older mosquitoes. Extending this model to incorporate other vector control interventions suggests that an IVM programme based on both ATSB and LLINs may substantially reduce mosquito density and survival rates in this setting, thereby substantially reducing parasite transmission. This is predicted to exceed the impact of LLINs in combination with IRS provided ATSB feeding rates are 50% or more of Mali experimental levels. In addition, ATSB is predicted to be particularly effective against *Anopheles arabiensis*, which is relatively exophilic and therefore less affected by IRS and LLINs.

**Conclusions:**

These results suggest that high coverage with a combination of LLINs and ATSB could result in substantial reductions in malaria transmission in this setting. Further field studies of ATSB in other settings are needed to assess the potential of ATSB as a component in future IVM malaria control strategies.

## Background

In the last decade, declines in the incidence of *Plasmodium falciparum* malaria have been reported throughout sub-Saharan Africa, occurring concomitantly with the extensive scale-up of insecticide-based vector control and the switch to artemisinin-based combination therapy (ACT) as first-line treatment [1-3]. Vector control strategies have largely focused on interventions which attack the vector indoors, in particular the use of long-lasting insecticide-treated nets (LLINs) and indoor residual spraying (IRS) with insecticides [4,5]. These are sometimes accompanied by efforts to control vector breeding sites through either source reduction or the application of larvicides [6]. This has resulted in substantial reductions in transmission and disease in many areas; however, in other areas, the reductions have been more modest [7]. This is partly due to the geographical variation in transmission potential which makes widespread elimination of the parasite difficult; however, there is also evidence that a residual population of outdoor-biting vectors, not targeted by indoor control measures, are able to sustain the parasite [8,9]. Thus it is clear that new vector control tools will be needed to maintain the recent gains made. Furthermore, these tools are essential in the face of evolving drug-resistance among parasites and insecticide-resistance among vectors [10].

Toxic sugar baits have been proposed as a novel vector control strategy that complements existing tools such as LLINs and IRS [11,12]. The strategy works by an “attract and kill” principle whereby mosquitoes are attracted to the fruity or flowery scent of the bait, and are then provided with a combination of sugar and an oral toxin such as boric acid, which is highly toxic to *Anopheles gambiae*, the primary African malaria vector [13,14]. The strategy has been extensively tested in Israel to suppress populations of the mosquito species *Anopheles sergentii*, *Anopheles claviger*, *Aedes caspius* and *Culex pipiens*[15-18] and has recently been tested in Bandiagara, a semi-arid area of Mali, to decimate populations of the malaria vector *An. gambiae s.l*. [14]. In Mali, ATSB solution sprayed onto vegetation near breeding sites was successful in reducing local vector densities by 90%, with the majority of remaining female mosquitoes being too young to transmit malaria. The strategy is, therefore, highly promising for malaria control in semi-arid areas of Africa, with further testing planned to determine its wider applicability.

A major benefit of ATSB is that, unlike LLINs and IRS, it targets female and male mosquitoes while they are outdoors. Larviciding is another important outdoor intervention, but is of limited use in rural areas where it is difficult to identify and treat all potential breeding sites [6,19,20]. Outdoor transmission is of growing importance as evidence suggests that intensive indoor control measures are causing transmission to shift from the mostly indoor-biting *An. gambiae* to the outdoor-adapted *An. arabiensis*[5,8,21,22]. Furthermore, *An. gambiae* appears to be becoming increasingly adapted to outdoor biting in some areas [9]. ATSB is also cheap and environmentally friendly, and oral toxins are not affected by the problem of insecticide-resistance [23]. That said, it is advisable that multiple toxins be used in an operational ATSB formula [14]. Effort will be required to ensure adequate vegetation coverage, particularly in less arid locations; however, ATSB benefits from the fact that sugar-feeding is a frequent behaviour for both male and female mosquitoes, and the sole food source for males [24,25].

This paper provides a quantitative basis for understanding the potential utility of ATSB as part of an integrated vector management (IVM) programme in Africa. Using results from the Mali field trial described earlier [14], mathematical models of sugar-feeding behaviour are fitted to the data to estimate parameters underlying the effectiveness of ATSB as a vector control strategy, including the rate of feeding on ATSB-sprayed plants and the expected lifetime of mosquitoes in the field following ingestion of the toxin. These parameters and an ecological model of *An. gambiae* and *An. arabiensis* dynamics are then used to investigate the impact of ATSB, as part of an IVM programme, on vector abundance and malaria transmission. The impact of a variety of vector control strategies on malaria transmission has been widely studied using mathematical models [26-30]; however, this study represents the first mathematical evaluation of the performance of ATSB, a highly promising, novel vector control strategy.

## Methods

### Trial data

Data were analysed from the above-mentioned ATSB field trial conducted near Bandiagara, Mali [14]. Two sites were monitored in this trial – an experimental site where ATSB was administered, and a control site where attractive (non-toxic) sugar bait (ASB) was used. Male and female catch numbers were recorded for six light traps at each site over a one-week pretreatment period and for 30 days post-treatment. The proportion of marked mosquitoes was also recorded, as in these experiments a coloured food dye that can be detected for several days after feeding was added to both ATSB and ASB solutions. To estimate age distribution among female mosquitoes, the number of gonotrophic cycles completed was recorded for a sample of 200 mosquitoes before and after the intervention, for both the experimental and control sites.

### Basic model selection

A range of simple, deterministic models were fitted to the Mali data. These included models with and without decay of dye, models in which sugar-feeding rates were the same or different in the control and experimental settings, and models in which mosquito emergence was assumed to be constant or proportional to the population size (Additional file 1). For each model, posterior parameter distributions were estimated using a Markov Chain Monte Carlo (MCMC) sampling procedure, and the deviance information criterion (DIC) was calculated as a measure for model selection (Additional file 2: Table S1). The best model was characterized by different sugar-feeding rates in the two settings, no decay of dye and a constant rate of mosquito emergence. For this model, the equations for female mosquitoes in the experimental setting are,

(1) $$\frac{dU_{E}}{\mathrm{dt}}=bN_{E}-s_{E}U_{E}-\mu U_{E}$$

(2) $$\frac{dM_{E}}{\mathrm{dt}}=s_{E}U_{E}-\mu_{\mathrm{ATSB}}M_{E}$$

Here, *U* and *M* represent the density of unmarked and marked female mosquitoes and the subscript *E* represents the experimental setting throughout (equivalently, the subscript *C* represents the control setting throughout). The adult emergence rate, *b*, is chosen to match the death rate, *μ*, so that the population is at equilibrium in the absence of ATSB. The equilibrium population size, as measured by mosquito catch numbers, is *N*. For the control setting, identical equations apply with the exception that marked mosquitoes are not exposed to the toxin and so also die at the rate *μ*. Equivalent equations hold for males. The equations for this model can be solved, for the experimental setting, to give,

(3) $$U_{E}\left(t\right)=N_{E}\frac{1}{\mu +s_{E}}\left(\mu +s_{E}e^{-\left(\mu +s_{E}\right)t}\right)$$

(4) $$\begin{array}{l} M_{E}\left(t\right)=N_{E}\frac{s_{E}}{\mu_{\mathrm{ASTSB}}\left(\mu_{\mathrm{ATSB}}-\mu -s_{E}\right)\left(\mu +s_{E}\right)}\\ \times \left(\begin{array}{l} \mu_{\mathrm{ATSB}}s_{E}e^{-\left(\mu +s_{E}\right)t}-\mu \left(\mu -\mu_{\mathrm{ATSB}}+s_{E}\right)\\ +\left(\mu -\mu_{\mathrm{ATSB}}\right)(\mu +s_{E})e^{-\mu_{\mathrm{ATSB}}t} \end{array}\right) \end{array}$$

Similarly, for the control setting, the equations are,

(5) $$U_{C}\left(t\right)=N_{C}\frac{1}{\mu +s_{C}}\left(\mu +s_{C}e^{-\left(\mu +s_{C}\right)t}\right)$$

(6) $$M_{C}\left(t\right)=N_{C}\frac{s_{C}}{\mu +s_{C}}\left(1-e^{-\left(\mu +s_{C}\right)t}\right)$$

For a given set of parameter values, an expression for the model likelihood can be derived by assuming the observed mosquito catch numbers are sampled from a negative binomial distribution with mean equal to the model-predicted mosquito density and variance to be estimated. A normal prior was used for daily mosquito mortality *μ*, with a mean of 0.1 per day and a standard deviation of 0.01 per day [31]. Uninformative uniform priors were used for all other model parameters. Posterior parameter distributions were estimated using an MCMC sampling procedure (Additional file 1).

### Model incorporating gonotrophic cycles

To accommodate the gonotrophic cycle data, the basic model (Equations 1, 2) was partitioned into unmarked females in the experimental setting, *U*_*i,E*_, and marked females in the experimental setting, *M*_*i,E*_, having completed *i* gonotrophic cycles, where *i∈* {0,1,…,8} (mosquitoes having completed eight or more cycles were grouped into the same category). Four models were then postulated to describe how the sugar-feeding rate may vary with cycle number: (i) feeding rate remains constant; (ii) feeding rate changes by a constant amount per cycle; (iii) feeding rate changes by a constant fraction per cycle; and, (iv) a step model in which feeding rate differs for mosquitoes having completed zero to two or three or more cycles (for more information, see Additional file 1). Once again, the DIC was used as a measure for model selection (Additional file 3: Table S2). The step model provided the best fit to the data and, for this model, the sugar-feeding rates vary with cycle number as,

(7) $$s_{i}=\left\{\begin{array}{l} s_{0},i\in \left\{0,1,2\right\}\\ ms_{0},i\geq 3 \end{array}\right)$$

Here, *s*_*i*_ is the sugar-feeding rate for a female mosquito having completed *i* gonotrophic cycles, and *m* is the fractional change in sugar-feeding rate for mosquitoes having completed three or more cycles (as compared to those having completed 0–2 cycles). Age-dependency of the mosquito death rate was considered, however a constant death rate was chosen because: (a) the pre-intervention gonotrophic cycle data is consistent with a constant death rate; (b) experimental data suggesting a higher death rate following initial emergence has not been confirmed under field conditions [32,33]; and, (c) a constant death rate leads to conservative predictions of disease transmission since an elevated death rate following emergence shifts the age distribution towards younger mosquitoes unable to transmit disease [34]. In the experimental setting, the model equations are given by,

(8) $$\frac{dU_{0,E}}{\mathrm{dt}}=bN_{E}-\left(s_{0,E}+\mu +\delta \right)U_{0,E}$$

(9) $$\frac{dM_{0,E}}{\mathrm{dt}}=s_{0,E}U_{0,E}-\left(\mu_{\mathrm{ATSB}}+\delta \right)M_{0,E}$$

(10) $$\frac{dU_{i,E}}{\mathrm{dt}}=\delta U_{i-1,E}-\left(s_{i,E}+\mu +\delta \right)U_{i,E},i\in \left\{1,\ldots ,7\right\}$$

(11) $$\frac{dM_{i,E}}{\mathrm{dt}}=\delta M_{i-1,E}+s_{i,E}U_{i,E}-\left(\mu_{\mathrm{ATSB}}+\delta \right)M_{i,E},i\in \left\{1,\ldots ,7\right\}$$

(12) $$\frac{dU_{8,E}}{\mathrm{dt}}=\delta U_{7,E}-\left(s_{8,E}+\mu \right)U_{8,E}$$

(13) $$\frac{dM_{8,E}}{\mathrm{dt}}=\delta M_{7,E}+s_{8,E}U_{8,E}-\mu_{\mathrm{ATSB}}M_{8,E}$$

Here, *δ* represents the reciprocal of the gonotrophic cycle length. The schematic for this model is shown in Additional file 4: Figure S1. Analogous equations apply in the control setting, replacing the subscript *E* with the subscript *C*. Analytic solutions to these equations are not feasible and so the differential equations must be solved numerically in order to compare the model predictions to the data.

Once again, an MCMC sampling procedure was used to estimate the posterior distributions of each of the model parameters. The likelihood function used was the same as for the basic models, multiplied by a term accounting for the comparison between the model-predicted and observed distribution of gonotrophic cycle number (Additional file 1). A normal prior was used for the parameter *δ*, with a mean of 0.33 per day and a standard deviation of 0.03 per day [34], and uninformative uniform priors were used for all other parameters.

### Model of integrated vector management

The IVM model divides the mosquito life cycle into larval, pupal and adult stages, thus allowing stage-specific interventions to be modelled [35]. Density-dependence is modelled at the larval stage, based on a study in Tanzania suggesting a linear relationship between larval density and mortality [36]. Parameters were estimated from the entomological literature and the Garki Project, undertaken in the 1970s in the Garki District of Nigeria (Additional file 5: Table S3). With this framework in place, a variety of interventions were simulated in isolation and synchrony to calculate their expected effects on *An. gambiae* and *An. arabiensis* densities.

The EIR for a particular setting was derived by multiplying the human biting rate (the number of bites per person per year) by the sporozoite rate, *S* (the proportion of the vector population that is infectious for malaria). The sporozoite rate was calculated by averaging over the gonotrophic cycle number, i.e.,

(14) $$S=\sum_{i}f_{i}S_{i}$$

Here, *f*_*i*_ represents the fraction of the female vector population having completed *i* gonotrophic cycles, and *S*_*i*_ represents the sporozoite rate of a female having completed *i* cycles. The sporozoite rate was calculated as a linearly-increasing function of cycle number accounting for the minimum number of cycles, *σ*, required for ingested parasites to become infectious in a mosquito [34],

(15) $$S_{i}=\left\{\begin{array}{l} 0,i\leq \sigma \\ \kappa Q_{0}\left(i-\sigma \right),i>\sigma \end{array}\right)$$

Here, *κ* represents the probability that a vector becomes infectious per human bite, assuming it survives long enough, and *Q*_0_ represents the proportion of blood-meals taken on humans in the absence of LLINs and IRS. Three transmission settings were considered with preintervention EIRs of 100 (very high transmission), 50 (high transmission) and 10 (moderate transmission). The human biting rate was varied according to the setting, and was consistent with estimates from Nigeria and Tanzania for the very high transmission setting [37-39]. Parameter estimates and their sources are included in Additional file 5: Table S3 and Additional file 6: Table S4.

## Results

### Estimates of exposure to ATSB and its impact on mortality using simple models

**Figure 1:**
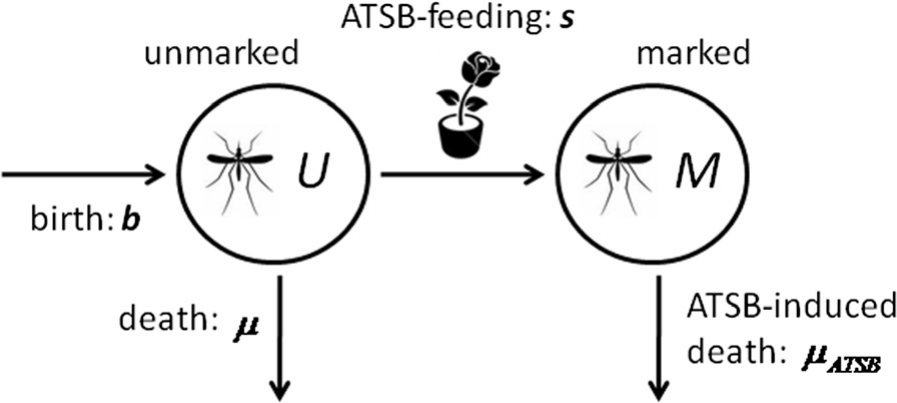
**Schematic of the basic ATSB sugar-feeding model in the experimental setting.** Female and male mosquitoes emerge at rate *b* into the unmarked class, *U*, and become marked, *M*, when feeding on ATSB-sprayed vegetation at rate *s*. In the control setting, marked and unmarked mosquitoes die at the same rate, *μ*, while in the experimental setting, marked mosquitoes die at a faster rate due to the effects of the toxin, *μ*_*ATSB*_.

The best-fitting sugar-feeding model was one in which there are two classes of mosquitoes – marked and unmarked. In this model, after emergence from pupae, female and male mosquitoes are unmarked and become marked when feeding on ASB or ATSB-sprayed vegetation. In the control setting, marked and unmarked mosquitoes die at the same rate, while in the experimental setting, marked mosquitoes die at a faster rate due to the effect of the toxin. A schematic for this model is shown in Figure 1.

**Figure 2:**
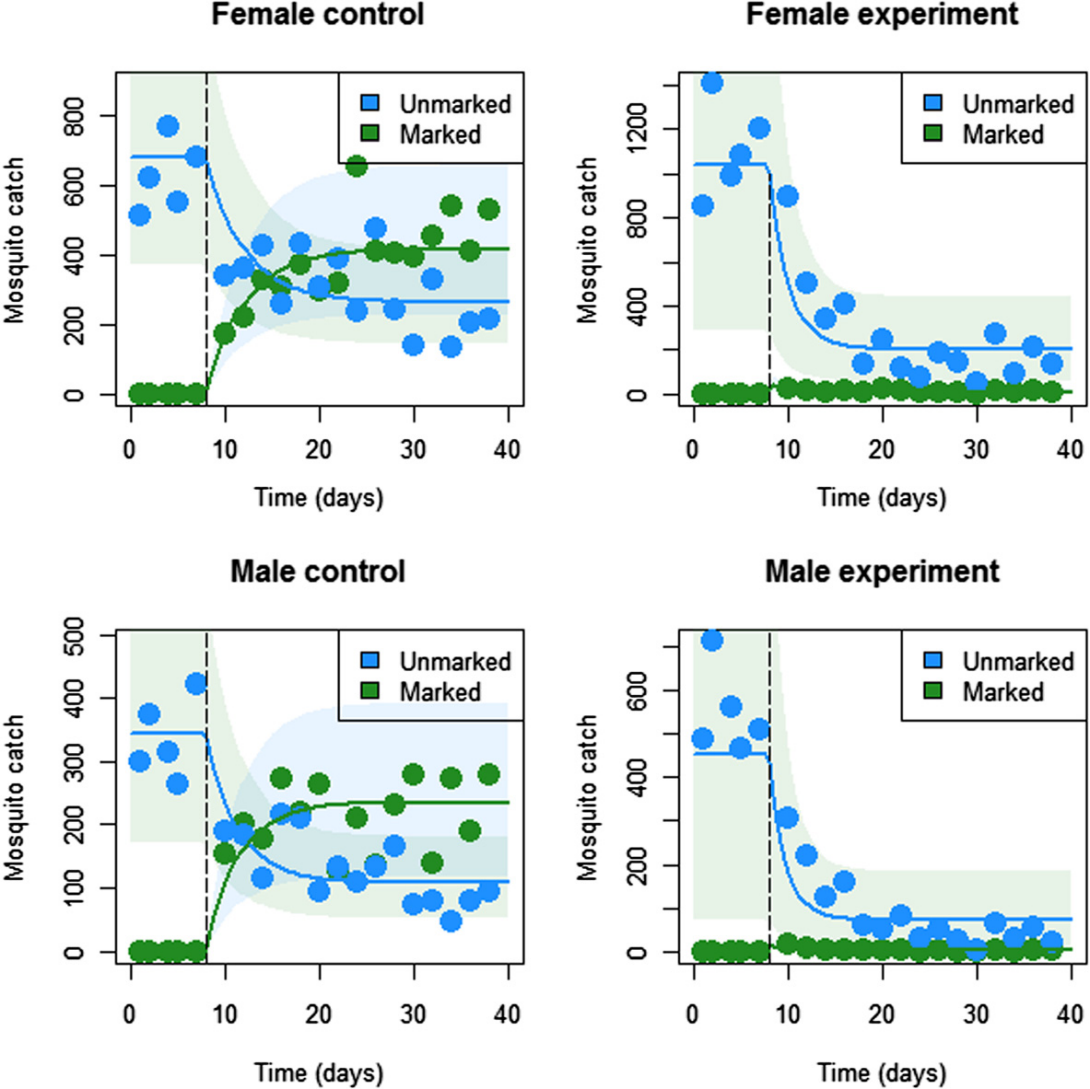
**Basic model fits for both male and female mosquito catch data in the experimental and control settings.** Dots represent mosquito catches, solid lines represent model predictions and shaded regions represent 95% of the model predicted variation in mosquito catch numbers.

Figure 2 depicts model fits for both male and female mosquito catches in the experimental and control settings with associated parameter estimates summarized in Table 1. Visually, the model provides a good fit to the data; although the estimated variation in mosquito catch data is somewhat large. Of most interest are the estimates of sugar-feeding rates and death rates upon ingesting the toxin. These are summarized in Table 1 along with 95% credible intervals (CrIs).

**Table 1:** Parameter estimates for basic sugar-feeding model

**Parameter:**	**Prior distribution (per day):**	**Posterior estimate with 95% credible interval (per day):**
Female ASB-feeding rate (control): *s*_*f*,*C*_	Uniform (0,10)	0.15 (0.12 - 0.19)
Female ATSB-feeding rate (experiment): *s*_*f*,*E*_	Uniform (0,10)	0.50 (0.27 - 0.97)
Male ASB-feeding rate (control): *s*_*m*,*C*_	Uniform (0,10)	0.15 (0.12 – 0.19)
Male ATSB-feeding rate (experiment): *s*_*m*,*E*_	Uniform (0,10)	0.46 (0.27 – 0.84)
Female death rate, *μ*_*f*_	Normal (0.1,0.01)	0.094 (0.075-0.115)
Male death rate, *μ*_*m*_	Normal (0.1,0.01)	0.094 (0.076-0.113)
Female ATSB death rate: *μ*_*f*__*,ATSB*_	Uniform (0,100)	11.7 (6.3 - 22.6)
Male ATSB death rate: *μ*_*m*__*,ATSB*_	Uniform (0,100)	11.0 (6.1 - 20.3)

The feeding rate of most relevance is that of females in the experimental setting, since only female mosquitoes bite and transmit malaria parasites. An ATSB feeding rate of 0.5 per female per day (95% CrI: 0.27-0.97) was estimated for the Mali experiment. Estimates of feeding rates differ significantly between the experimental and control settings (0.50 per day for the experimental setting versus 0.15 per day for the control setting) which could be due to differences in the relative abundance of sugar bait in the two settings (either in terms of application level or the availability of natural sugar sources), or due to dye decay causing the ASB-feeding rates to be underestimated (in the experimental setting, dye decay can be ignored since toxin-induced death occurs at a faster rate). Given that mosquitoes also feed on natural sugar sources, the total sugar-feeding rate will be higher than both of these estimates.

The death rates following ingestion of ATSB are important indicators of the effectiveness of ATSB at reducing mosquito density. For females, an estimated death rate of 11.7 per day corresponds to a mean lifetime of 2.1 hours following ATSB consumption (95% CrI: 1.1-3.8 hours). This estimate is consistent with laboratory experiments showing 100% lethality within 12 hours [14]. It should be noted that, while relevant to mosquito density, this parameter is less relevant to malaria control since mosquitoes tend not to seek blood meals after feeding on ATSB [13].

### Incorporating gonotrophic cycles

Female mosquitoes blood-feed to fuel the production of eggs. The number of blood-feeding and egg-laying (gonotrophic) cycles they have completed provides a measure of their age – each cycle takes approximately three days to complete [34] – and their ability to transmit pathogens. At the earliest, mosquitoes can become infected with malaria on their first gonotrophic cycle, and it takes at least another two cycles for the parasites to incubate within the mosquito [34]. This means that only female mosquitoes that have completed three or more gonotrophic cycles can be infectious to humans. Gonotrophic cycle numbers as high as eight were recorded in the Mali field trial [14] and these provide an opportunity to investigate trends in sugar-feeding with age.

**Figure 3:**
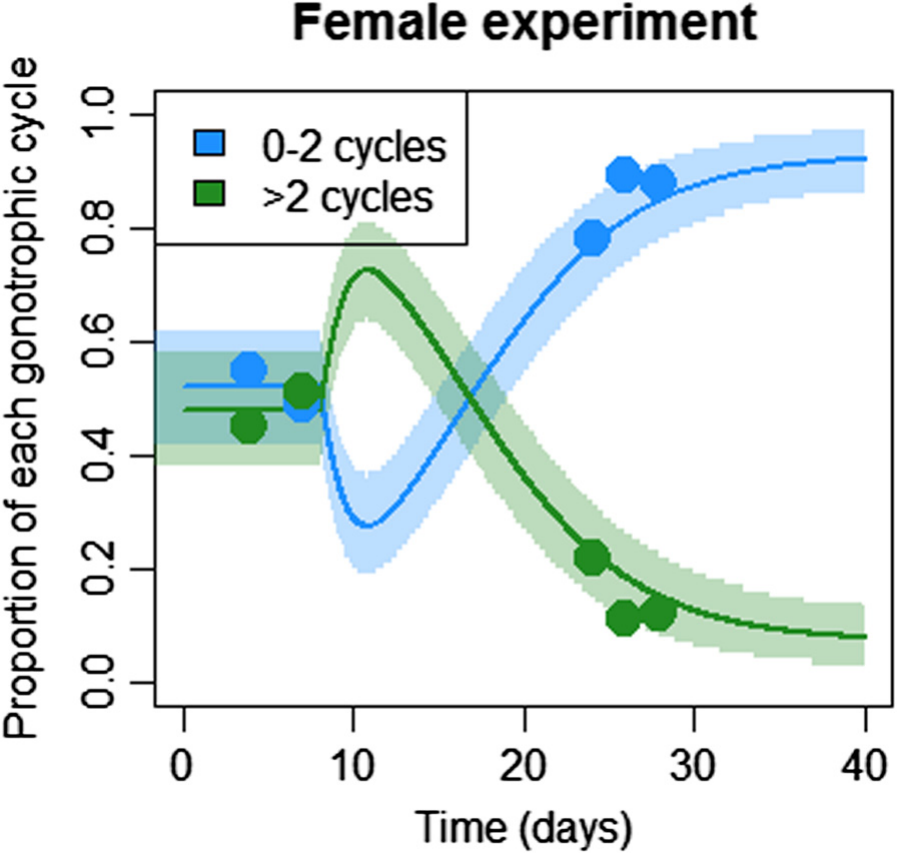
**Proportions of female mosquitoes having completed zero to two or more than two gonotrophic cycles in the experimental setting.** Dots represent observed results, solid lines represent model predictions and shaded regions represent 95% of the model-predicted variation in mosquito catch numbers. Although mosquito numbers in all categories decline after the addition of ATSB, females having completed zero to two gonotrophic cycles decline fastest initially because they have the highest sugar-feeding rate; however, the age distribution quickly shifts towards younger mosquitoes.

Figure 3 shows model fits for the proportion of female mosquitoes having completed 0–2 or more than two gonotrophic cycles in the experimental setting. The intervention was at day seven and the first post-intervention data regarding gonotrophic cycles was collected at day 24, hence there is limited power to predict changes in the breakdown of gonotrophic cycle numbers between these time points. However, it is clear that three weeks after the intervention, very few female mosquitoes remained that had completed more than two gonotrophic cycles. In the control site, the gonotrophic cycle number distribution remained constant over time.

Table 2 shows revised estimates of sugar-feeding rates and ATSB-induced death rates for a model in which mosquitoes having completed zero to two and three or more gonotrophic cycles have distinct sugar-feeding rates. This was the best-fitting of four models in which sugar-feeding rates were allowed to vary with age (Additional file 3: Table S2). Interestingly, for all four models, a significant reduction in sugar-feeding rate with age was observed. The model suggested a sugar-feeding rate for females having completed zero to two gonotrophic cycles that was almost double the mean sugar-feeding rate estimated from the simpler, non-age-structured model. Balancing this, the estimated sugar-feeding rate for females having completed three or more gonotrophic cycles was about a quarter the mean sugar-feeding rate. The feeding rate on ATSB-sprayed vegetation in the experimental setting of 0.84 per day is consistent with empirical evidence that young mosquitoes sugar-feed more than once per day [14], since the actual sugar-feeding rate is higher than that solely on ATSB-sprayed sources.

**Table 2:** Parameter estimates for sugar-feeding model incorporating gonotrophic cycles

**Parameter:**	**Prior distribution (per day):**	**Posterior estimate with 95% credible interval (per day):**
ASB-feeding rate (0–2 gonotrophic cycles, control): *s*_*A*,*C*_	Uniform (0,10)	0.25 (0.18 - 0.31)
ASB-feeding rate (3 or more gonotrophic cycles, control): *s*_*B*,*C*_	Uniform (0,10)	0.035 (0.009 – 0.076)
ATSB-feeding rate (0–2 gonotrophic cycles, experiment): *s*_*A*,*E*_	Uniform (0,10)	0.84 (0.53 – 1.21)
ATSB-feeding rate (3 or more gonotrophic cycles, experiment): *s*_*B*,*E*_	Uniform (0,10)	0.12 (0.03 – 0.27)
Female death rate, *μ*_*f*_	Normal (0.1,0.01)	0.094 (0.081-0.110)
Female ATSB death rate: *μ*_*f*__*,ATSB*_	Uniform (0,100)	12.2 (7.5 – 23.9)
Reciprocal of gonotrophic cycle length: δ	Normal (0.33,0.03)	0.34 (0.30-0.39)

### The potential impact of ATSB as part of integrated vector management (IVM)

**Figure 4:**
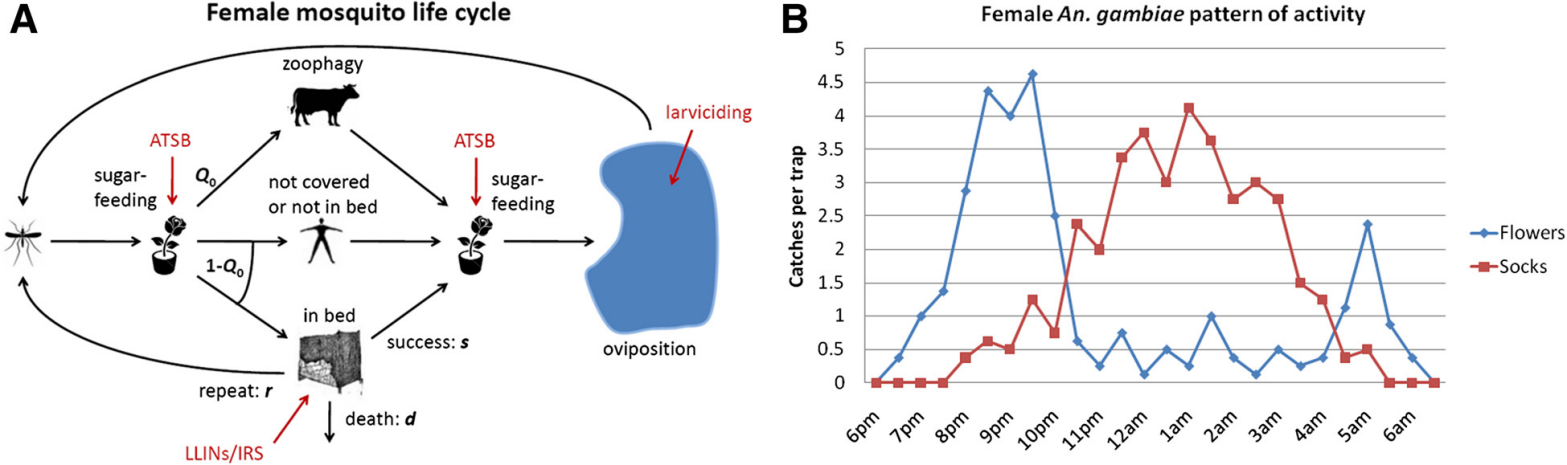
**Sugar-feeding and integrated vector management. A)** Life cycle of the female mosquito depicting the centrality of sugar-feeding and opportunities for vector control in red. **B)** Data on the pattern of activity of female *An. gambiae* mosquitoes. Mosquito catches on flowers peaked at 9 pm and 5 am, while catches on socks peaked around 1 am.

Current vector control strategies focus largely on LLINs and IRS; however both these interventions target adult mosquitoes while they are indoors. The addition of ATSB holds promise because it targets adult mosquitoes outdoors and also complements larviciding, which targets the aquatic stage of the mosquito life cycle (Figure 4A). Data on the pattern of mosquito activity (Figure 4B) also suggest that mosquitoes sugar-feed at different times to seeking a blood-meal – specifically, at dusk before blood-feeding, and, to a lesser extent, just before sunrise. This further highlights the potential synergy between ATSB and other vector control strategies.

To assess the potential contribution of ATSB to IVM strategies, the models and parameters described above were used in conjunction with an existing ecological model of *Anopheles* population dynamics [34] and an existing model [40] of the effects of LLINs and IRS on mosquito densities, modified slightly as in Griffin *et al.*[41]. For larviciding, the case of *Bacillus thuringiensis var. israelensis* (BTI) applied to larval breeding sites was considered [42]. BTI was found to reduce larval density by 88% where applied [42] and to increase larval and pupal death rates by a constant factor. Coverage levels for current vector interventions were assumed to be either 80% or 50% (Additional file 1), and ATSB was assumed to be implemented at levels leading to an exposure rate analogous to that in the Mali experimental setting [14] or at levels such that the exposure rate would be half that of the Mali setting. The combined model is described in Additional file 1.

**Figure 5:**
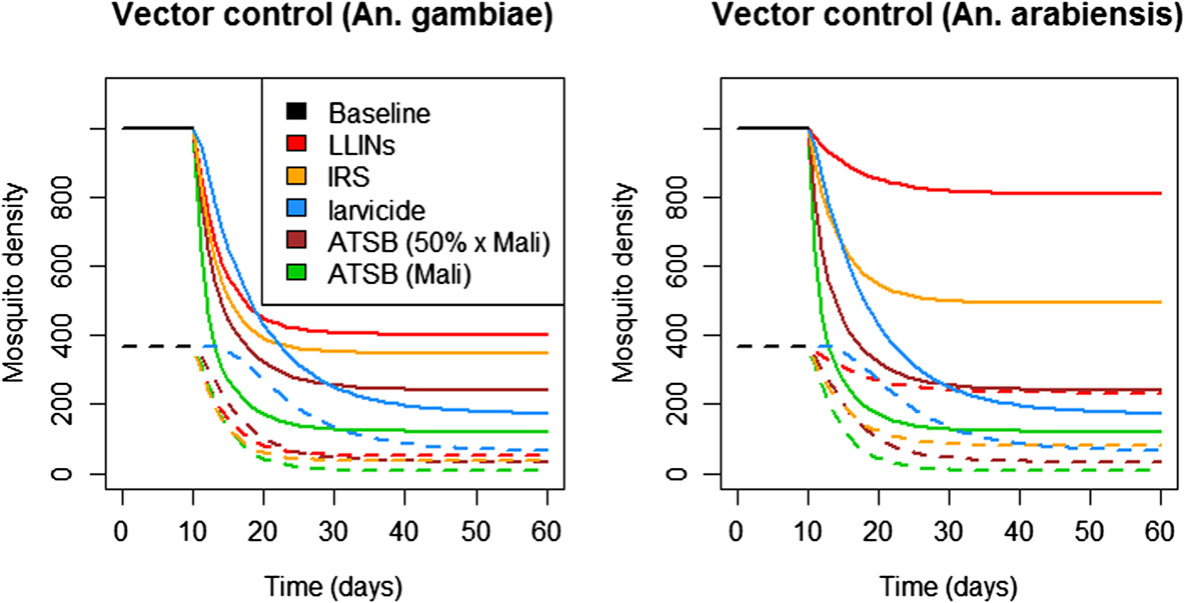
**Time-series depicting the effects of vector control strategies on vector density in isolation.** Solid lines represent total female mosquito density, dashed lines represent females having completed three or more gonotrophic cycles. Coverage levels are assumed to be 80% for all interventions (i.e. 80% of people sleeping under nets, 80% of houses sprayed with insecticide, and 80% of breeding sites treated with BTI). ATSB is assumed to be implemented at analogous levels to that in the Mali experimental setting, and at levels such that the exposure rate would be half that of the Mali experimental setting.

At 80% coverage, LLINs and IRS are expected to significantly reduce *An. gambiae* density (Figure 5). However, LLINs are expected to have less effect on the more exophilic *An. arabiensis*. Interestingly, at Mali exposure rates, ATSB is expected to have a greater population suppressing effect than either LLINs or IRS – a trend also seen if ATSB exposure rates are halved. All three of these interventions result in an age distribution heavily skewed towards females having completed two or less gonotrophic cycles, which is encouraging for malaria control. ATSB and larviciding perform similarly well at reducing adult mosquito densities; however, since larvicides act before the adult life stage, they don’t cause any changes in the adult age structure. If ATSB coverage is such that exposure rates are half those of the Mali experimental setting, larviciding has a bigger effect on the total mosquito density but a smaller effect on mosquitoes having completed three or more gonotrophic cycles. ATSB is therefore more efficient at reducing the number of mosquitoes that could potentially transmit malaria. If coverage with the other interventions is reduced to 50%, ATSB is predicted to outperform all of them even at 50% Mali exposure rates (Additional file 7: Figure S2).

**Figure 6:**
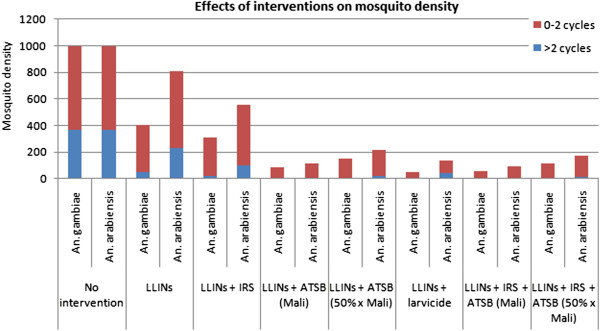
Expected impact of IVM strategies on mosquito density.

Figure 6 and Additional file 8: Figure S3 show the expected impact of different combinations of interventions on *An. gambiae* and *An. arabiensis* densities assuming a pre-intervention density of 1,000 for both species, 369 of which have completed three or more gonotrophic cycles. A combination of LLINs and ATSB is expected to be extremely efficient at reducing population densities of both species and, in particular, the density of female mosquitoes having completed three or more gonotrophic cycles (reduced to ~2 for *An. gambiae* and ~5 for *An. arabiensis*). The LLIN/ATSB combination compares favourably against a combination of LLINs and IRS or LLINs and larviciding, even when ATSB exposure rates are halved (in which case, the density of *An. gambiae* having completed three or more gonotrophic cycles is reduced to ~7, and to ~23 for *An. arabiensis*). For the LLIN/larviciding combination, mosquito densities are reduced; however there is still a residual *An. arabiensis* population with a density of ~39 having completed three or more gonotrophic cycles. Addition of IRS to the LLIN/ATSB combination provides little benefit due to the efficiency of the LLIN/ATSB combination on its own.

### Impact of IVM strategies including ATSB on EIR

Reductions in vector density give a clear comparison of the relative impact of IVM strategies; however a more direct measure of human exposure to malaria is the entomological inoculation rate (EIR), defined as the average number of infective bites per person per year [39]. The EIR is more sensitive to the age breakdown of the vector population, since older mosquitoes are more likely to be infectious to humans.

**Figure 7:**
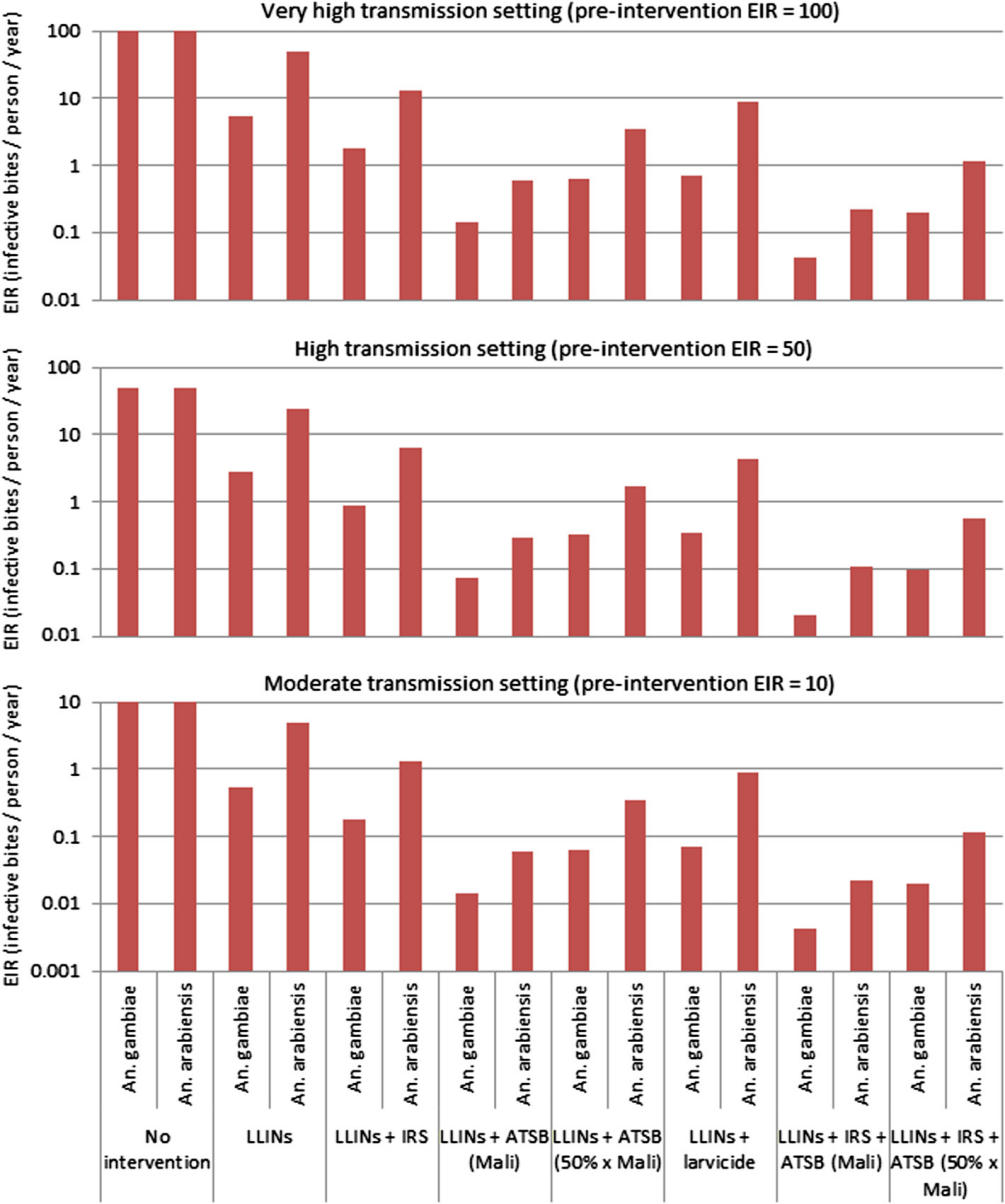
**Expected impact of IVM strategies on EIR.** Model predictions are shown for three transmission settings with pre-intervention EIRs of 100 (very high transmission), 50 (high transmission) and 10 (moderate transmission).

Figure 7 shows the expected impact of the same interventions as shown in Figure 6 on EIR for three transmission settings, and Additional file 9: Figure S4 shows these for 50% coverage levels with current interventions. For simplicity of comparison, populations are assumed to be entirely either *An. gambiae* or *An. arabiensis*. The relative impact of the different combinations of interventions is the same in each setting, although the magnitude of the post-intervention EIR differs. For 80% coverage levels in settings with baseline EIRs of 50 and 100, only the LLIN/ATSB combination (with Mali ATSB exposure rates) is expected to reduce EIRs to less than one infective bite per person per year for both species, which is the value thought necessary to achieve local elimination [43-45]. For 50% coverage levels and ATSB exposure rates at 50% of those in Mali, only the LLIN/ATSB combination is expected to reduce EIRs to less than one in the moderate transmission setting and to less than ten in the high and very high transmission settings. These results should not be interpreted as predictive; but they do suggest that ATSB could potentially play an important role in vector control in a range of transmission settings.

**Figure 8:**
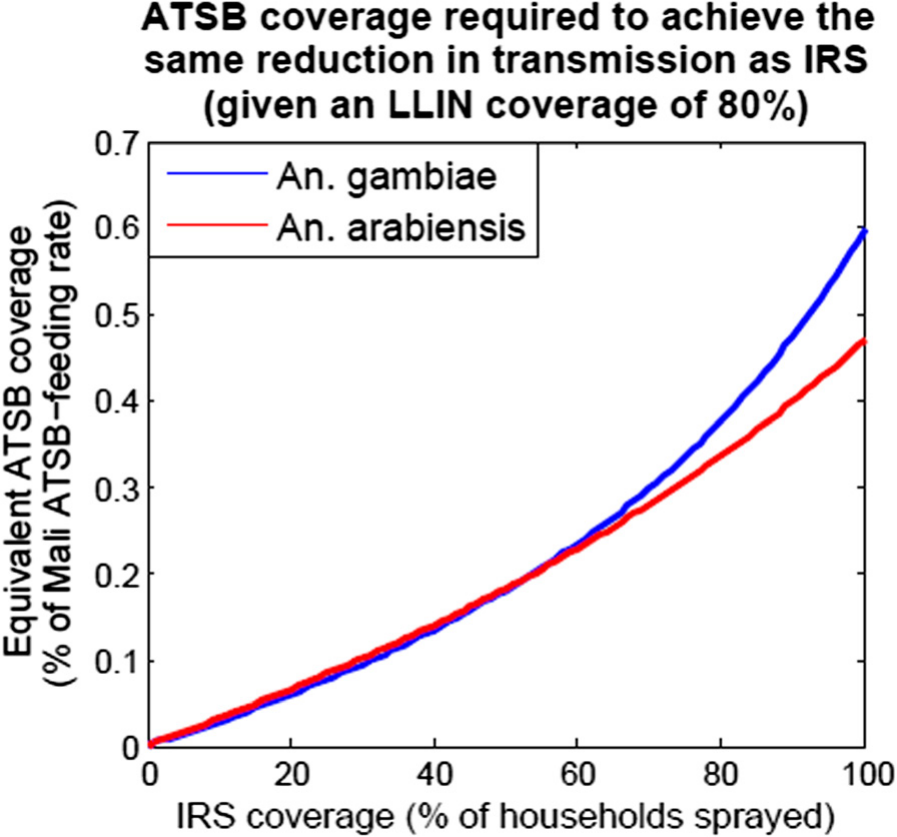
ATSB coverage required to achieve the same reduction in transmission as IRS (given an LLIN coverage of 80%).

A question of great relevance to IVM planning is the ATSB exposure rate at which a combination of LLINs and ATSB is expected to cause a larger reduction in malaria transmission (as measured by EIR) than a combination of LLINs and IRS. LLINs are now widely distributed in many malaria-endemic countries [4,7], and hence 80% LLIN coverage levels are assumed for these calculations. IRS is less widespread and more likely to be replaced by alternative interventions; however, before being replaced, the new intervention should be expected to be more effective at reducing malaria transmission than IRS when used in combination with the already-present LLINs. Figure 8 shows the ATSB exposure rates (measured as a fraction of Mali exposure rates) required to achieve the same reduction in EIR as IRS at a range of coverage levels between 0 and 100%. To compensate for IRS at an optimistic coverage level of 80%, modelling suggests that ATSB exposure rates of ~36% of Mali levels (34% for *An. arabiensis* and 38% for *An. gambiae*) would be required to achieve the same reduction in malaria transmission (these results are independent of the baseline EIR for this model). The lower requirement for *An. arabiensis* is due to it being relatively exophilic and hence more susceptible to outdoor control measures. The predicted effectiveness of ATSB coverage levels less than in the Mali experimental setting is encouraging; however, further experiments will be required to determine the relationship between coverage level and exposure rate in a range of environmental settings, including in lush settings with an abundance of natural sugar sources.

## Discussion

The promise of ATSB described here directly follows from extending the results of a successful field trial in Bandiagara, Mali [14] to a range of different transmission intensities, and modelling its impact in combination with a variety of other vector control strategies. The models suggest that high coverage with a combination of LLINs and ATSB at levels similar to those in the Mali field trial is expected to cause significant reductions in EIR, exceeding the predicted impact of LLINs in combination with other interventions such as IRS or larviciding. Furthermore, ATSB is expected to perform favourably even at half the exposure rates of the Mali field trial.

The benefit of ATSB is that it kills mosquitoes while they are outdoors, thus targeting a different stage of the mosquito gonotrophic cycle than LLINs and IRS [46]. Larviciding targets a different stage of the mosquito life cycle; however ATSB has the advantage that it skews the adult age distribution towards younger mosquitoes, which is beneficial for malaria control because only older mosquitoes have time to acquire, incubate and transmit the parasite. It is also cheap and environmentally friendly and, while not modelled here, it targets both male and female mosquitoes.

An interesting result from the model fits was a significant trend in declining sugar-feeding rate with age among female mosquitoes. Since older mosquitoes are more likely to transmit malaria, a strategy that targets these older mosquitoes is desirable; however, if mosquitoes are targeted when they are young, they will not reach the required age to transmit malaria, so both approaches are effective. This is evidenced by the scarcity of mosquitoes having completed more than two gonotrophic cycles within a few weeks of ATSB application in Mali (Figure 3). That said, it is not clear the extent to which this trend is influenced by the high rate of sugar-feeding following emergence [25]. Regardless, the results from this trial suggest a high death rate among young mosquitoes, which is predicted to reduce the number of adult mosquitoes capable of transmitting malaria similarly to strategies that target adult mosquitoes in an age-independent manner.

Also worthy of note is that the sugar-feeding rates estimated here are for ATSB and ASB-sprayed vegetation at the coverage levels of the Mali experiment. An estimate of the total sugar-feeding rate on all available vegetation would be of interest to understanding the maximum potential of ATSB at reducing mosquito density. One way to measure this would be to spray patches of vegetation with ASB containing different coloured dyes. Coloured and multicoloured mosquitoes could then be used to infer the total sugar-feeding rate in a similar manner to how total population size is inferred in a traditional mark-release-recapture experiment. Also of interest is the relationship between coverage level and ATSB-feeding rate. In the Mali trial, one square metre spots of vegetation were sprayed every three metres around breeding sites [14] leading to the ATSB-feeding rates estimated here. A relationship between these variables would assist in operational and cost-effectiveness analyses.

The performance of ATSB in different geographic and seasonal settings is of great interest. Field trials have thus far been conducted in Mali and Israel [14-18] and provide a proof of principle in semi-arid areas. In Israel, ATSB has been shown to outcompete natural sugar sources [47] and to reduce mosquito populations even in sugar-rich environments [48]. Modelling results presented here predict ATSB to be effective even if exposure rates are half those of the Mali experiment. However, the performance of ATSB remains to be tested in settings with a greater abundance of natural sugar sources. A further complication is that heavy rains can wash ATSB off of vegetation, making reapplication necessary during the rainy season. A complementary approach is the provision of covered bait stations, which have proven successful in Israel [15,16], and are currently undergoing field testing and product development in other settings. Another product enhancement being considered is combining ATSB with larvicides which mosquitoes may carry to breeding sites after sugar-feeding.

As for any modelling exercise, simplifications have been made and limitations exist that mean that the results are indicative rather than predictive. The sugar-feeding model is parameterized by fitting to the available trial data from one semi-arid location; however, the parameter estimates include a high degree of uncertainty. Furthermore, the true underlying dynamics may be more complicated than suggested by the simple parsimonious models explored here. For instance, sugar-feeding rates are likely to decline more gradually with age than was possible to detect by fitting to the available data and dye decay was not incorporated but is known to occur in the wild [14]. However, the general trends inferred here capture important features of vector control with ATSB. Parameter estimates for other vector control strategies are collated from several different locations and neglect phenomena such as waning of efficiency with time. Whilst the LLIN model captures the effect size observed in randomized trials, both the IRS and larviciding models have not been validated against trial data. Furthermore, *An. gambiae* and *An. arabiensis* have been considered as separate entities here, while future studies could investigate potential shifts in species composition under a variety of IVM combinations using a species competition model [49]. Therefore, the IVM model predictions should be interpreted in this light as providing insight into the potential of ATSB to contribute to future integrated vector control programs rather than precise predictions.

## Conclusions

In summary, the models presented suggest that ATSB, or modifications of this approach to target outdoor mosquitoes, could be important to consider in future IVM programmes, especially in combination with LLINs and in semi-arid areas. ATSB kills mosquitoes while they are outdoors and skews the adult age distribution towards younger mosquitoes, leading to substantial reductions in both sporozoite rate and EIR. Further field testing is needed to address operational issues (in particular the degree of overall coverage that can be obtained) and to determine its efficacy in a range of other settings. If the predictions of this modelling effort hold true, ATSB could be a useful additional tool for malaria control in permissive settings.

## Competing interests

The authors declare that they have no competing interests.

## Authors’ contributions

JMM, MTW, GCM and JCM devised the study and objectives. GCM provided the data. JMM and MTW developed the model and analysed the data. JMM wrote the first draft of the manuscript. All authors read and approved the final manuscript.

## Supplementary Material

Additional file 1 Further details on sugar-feeding and integrated vector management models, model fitting and parameter values. Click here for file

Additional file 2: Table S1 Model comparison for basic models. Click here for file

Additional file 3: Table S2 Model comparison for models incorporating gonotrophic cycle number. Click here for file

Additional file 4: Figure S1 Schematic of sugar-feeding model incorporating gonotrophic cycles in the experimental setting. Umarked, *U*_*i*_, and marked, *M*_*i*_, females are partitioned into those having completed *i* gonotrophic cycles, where *i*ϵ{0,1,…,8} (mosquitoes having completed eight or more cycles are grouped into the same category). Female mosquitoes emerge at rate *b* into unmarked class, *U*_0_, and become marked, *M*_0_, when feeding on ATSB-sprayed vegetation at rate *s*_*i*_. In the control setting, marked and unmarked mosquitoes die at the same rate, *μ*, while in the experimental setting, marked mosquitoes die at a faster rate due to the effects of the toxin, ^μ^_*ATSB*_. Both marked and unmarked mosquitoes have a gonotrophic cycle length of 1/*δ*. Click here for file

Additional file 5: Table S3 Parameter estimates for IVM model that are species-invariant. Click here for file

Additional file 6: Table S4 Parameter estimates for IVM model that vary between species. Click here for file

Additional file 7: Figure S2 Time-series depicting the effects of vector control strategies on vector density in isolation. Solid lines represent total female mosquito density, dashed lines represent females having completed three or more gonotrophic cycles. Coverage levels are assumed to be 50% for all interventions (i.e. 50% of people sleeping under nets, 50% of houses sprayed with insecticide, and 50% of breeding sites treated with BTI). ATSB is assumed to be implemented at analogous levels to that in the Mali experimental setting, and at levels such that the exposure rate would be half that of the Mali experimental setting. Click here for file

Additional file 8: Figure S3 Expected impact of IVM strategies on mosquito density. Red bars represent females having completed less than three gonotrophic cycles and blue bars represent females having completed three or more gonotrophic cycles. Coverage levels are assumed to be 50% for all interventions (i.e. 50% of people sleeping under nets, 50% of houses sprayed with insecticide, and 50% of breeding sites treated with BTI). ATSB is assumed to be implemented at analogous levels to that in the Mali experimental setting, and at levels such that the exposure rate would be half that of the Mali experimental setting. Click here for file

Additional file 9: Figure S4 Expected impact of IVM strategies on EIR. Coverage levels are assumed to be 50% for all interventions (i.e. 50% of people sleeping under nets, 50% of houses sprayed with insecticide, and 50% of breeding sites treated with BTI). ATSB is assumed to be implemented at analogous levels to that in the Mali experimental setting, and at levels such that the exposure rate would be half that of the Mali experimental setting. Model predictions are shown for three transmission settings with pre-intervention EIRs of 100 (very high transmission), 50 (high transmission) and 10 (moderate transmission). Click here for file
