# Supplementary material for: Quantifying the mosquito’s sweet tooth: modelling the effectiveness of attractive toxic sugar baits (ATSB) for malaria vector control
Source: Malar J. 2013 Aug 23;12:291. doi: 10.1186/1475-2875-12-291 (PMC3765557; doi:10.1186/1475-2875-12-291)
Supplement: Additional file 1 — Further details on sugar-feeding and integrated vector management models, model fitting and parameter values. [file 1475-2875-12-291-S1.pdf]

Additional file 1

Text

Basic Model Selection. Six models were fitted to the Mali data. These included models with and without decay of dye, models in which sugar-feeding rates are the same or different in the control and experimental settings, and models in which mosquito emergence was assumed to be constant or proportional to the population size. The deviance information criterion (DIC) was used as a measure for model selection (Table S1), the best model being characterized by different sugar-feeding rates in the two settings, no decay of dye and a constant rate of mosquito emergence. Its equations and their solutions are described in Equations 1-6. The equations for the other five models are described here.

For the model in which sugar-feeding rates are assumed to be the same in both settings, dye decay is ignored and mosquito emergence is constant, Equations 1-6 continue to apply with the modification that there is now a common sugar-feeding rate, $s = s_C = s_E$. Here, the subscripts *C* and *E* represent the control and experiental settings, respectively, throughout. If dye decay is not ignored in the control setting, then the equations for this setting become,

$$\frac{dU_C}{dt} = bN_C - sU_C - \mu U_C + \rho M_C \ , \qquad \text{(S1)}$$

$$\frac{dM_C}{dt} = s_C U_C - \mu M_C - \rho M_C \ . \qquad \text{(S2)}$$

As before, *U* and *M* represent the density of unmarked and marked female mosquitoes. The adult emergence rate, *b*, is chosen to match the death rate, μ, so that the population is at equilibrium in the absence of ATSB. The equilibrium population size, as measured by mosquito catch numbers, is *N*, and the dye decay rate is ρ. The equations for this model can be solved, for the experimental setting, to give,

$$U_C(t) = N_C \frac{1}{\mu + s + \rho} e^{-(\mu+s+\rho)t}((\mu+\rho)e^{(\mu+s+\rho)t} + s) \ , \qquad \text{(S3)}$$

$$M_C(t) = N_C \frac{s}{\mu + s + \rho} e^{-(\mu+s+\rho)t}(e^{(\mu+s+\rho)t} - 1) \ . \qquad \text{(S4)}$$

For the remaining three models, mosquito emergence is proportional to the total mosquito population size. This more closely represents reality since the emergence rate declines as the population size declines, although it neglects density-dependence at the larval stage. For the model in which sugar-feeding rates differ between the control and experimental settings and dye decay is ignored, Equations 5-6 continue to describe the control setting since the total population size remains constant and hence emergence remains constant; however, for the experimental setting, Equations 1-2 become,

$$\frac{dU_E}{dt} = b(U_E + M_E) - s_E U_E - \mu U_E \ , \tag{S5}$$

$$\frac{dM_E}{dt} = s_E U_E - \mu_{ATSB} M_E \ . \tag{S6}$$

These equations can be solved to give,

$$U_E(t) = N_E \frac{1}{z} e^{-\frac{1}{2}(\mu_{ATSB}+s_E)t} \left(z\cosh\left(\frac{1}{2}zt\right) + (\mu_{ATSB} - s_E)\sinh\left(\frac{1}{2}zt\right) \right) , \tag{S7}$$

$$M_E(t) = N_E \frac{s_E}{z} e^{-\frac{1}{2}(\mu_{ATSB}+s_E+z)t} \left(e^{zt} - 1\right) , \tag{S8}$$

where,

$$z = \sqrt{\mu_{ATSB}^2 - 2\mu_{ATSB} s_E + s_E(4\mu + s_E)} \ . \tag{S9}$$

For the equivalent model in which sugar-feeding rates are assumed to be the same in both settings, Equations S7-S9 continue to apply with the substitution of a common sugar-feeding rate $s = s_C = s_E$. Finally, if dye decay is not ignored in the control setting, then Equations S1-S4 describe the control setting while Equations S7-S8 continue to describe the experimental setting.

Model Incorporating Gonotrophic Cycles. Four models were postulated to describe how the sugar-feeding rate may vary with cycle number. First, a model was considered in which the sugar-feeding rate remains constant – i.e. $s_i = s_0$, or in other words, the sugar-feeding rate for a mosquito having completed *i* gonotrophic cycles, s_i, is the same as the baseline sugar-feeding rate, s_0. A model was then considered in which the sugar-feeding rate changes by a constant amount, *m*, with each additional gonotrophic cycle, i.e.

$$s_i = s_0(1 + im) \ . \tag{S10}$$

Thirdly, a model was considered in which the sugar-feeding rate changes by a constant fraction, *m*, with each additional gonotrophic cycle, i.e.

$$s_i = s_0 m^i \ . \tag{S11}$$

Finally, a step model was considered in which the sugar-feeding rate differs for mosquitoes having completed 0-2 or three or more cycles (Equation 7). The DIC value was used as a measure for model selection (Table S2), the best model being the step model described by Equation 7.

Model Fitting. To determine the parameter values that provide the best fit to the data, a Bayesian approach with relatively uniformative priors was used. Assuming a Negative Binomial

distribution in mosquito catch numbers, for the basic models neglecting the gonotrophic cycle number, the data likelihood is given by:

$$L_1(N_C, N_E, s_C, s_E, \mu, \mu_{ATSB}, r_C, r_E) =$$
$$\prod_k \binom{u_C(k)+r_C-1}{u_C(k)} \times \left(1-\frac{U_C(k)}{U_C(k)+r_C}\right)^{r_C} \left(\frac{U_C(k)}{U_C(k)+r_C}\right)^{u_C(k)}$$
$$\times \prod_k \binom{m_C(k)+r_C-1}{m_C(k)} \times \left(1-\frac{M_C(k)}{M_C(k)+r_C}\right)^{r_C} \left(\frac{M_C(k)}{M_C(k)+r_C}\right)^{m_C(k)} \quad . \quad \text{(S12)}$$
$$\times \prod_k \binom{u_E(k)+r_E-1}{u_E(k)} \times \left(1-\frac{U_E(k)}{U_E(k)+r_E}\right)^{r_E} \left(\frac{U_E(k)}{U_E(k)+r_E}\right)^{u_E(k)}$$
$$\times \prod_k \binom{m_E(k)+r_E-1}{m_E(k)} \times \left(1-\frac{M_E(k)}{M_E(k)+r_E}\right)^{r_E} \left(\frac{M_E(k)}{M_E(k)+r_E}\right)^{m_E(k)}$$

Here, k represents the time points at which data has been collected, $m(k)$ and $u(k)$ represent the measured marked and unmarked mosquito catch sizes at these time points and $M(k)$ and $U(k)$ represent the model predictions as described above. Parameters requiring estimation are N_C, N_E, s_C, s_E, μ, μ_{ATSB}, r_C and r_E, where r is a shape parameter describing the level of overdispersion in mosquito catch numbers. A Normal prior was used for the mosquito death rate parameter, μ and uniformative Uniform priors for all other parameters.

For models including the gonotrophic cycle number, the same likelihood function was used as described in Equation S12 and multiplied this by a term accounting for the gonotrophic cycle number (assuming a multinomial distribution in cycle number),

$$L_2(N_C, N_E, s_C, s_E, \mu, \mu_{ATSB}, r_C, r_E, \delta.m) =$$
$$L_1(N_C, N_E, s_C, s_E, \mu, \mu_{ATSB}, r_C, r_E, \delta, m)$$
$$\times \prod_k \frac{n_C(k)!}{n_{1,C}(k)!...n_{8,C}(k)!} \times \left(\frac{U_{1,C}(k)+M_{1,C}(k)}{U_C(k)+M_C(k)}\right)^{n_{1,C}(k)} ... \left(\frac{U_{8,C}(k)+M_{8,C}(k)}{U_C(k)+M_C(k)}\right)^{n_{8,C}(k)} \quad . \quad \text{(S13)}$$
$$\times \prod_k \frac{n_E(k)!}{n_{1,E}(k)!...n_{8,E}(k)!} \times \left(\frac{U_{1,E}(k)+M_{1,E}(k)}{U_E(k)+M_E(k)}\right)^{n_{1,E}(k)} ... \left(\frac{U_{8,E}(k)+M_{8,E}(k)}{U_E(k)+M_E(k)}\right)^{n_{8,E}(k)}$$

Here, k represents the time points at which gonotrophic data was collected, $n(k)$ represents the number of female mosquitoes that were age-graded at time k, $n_i(k)$ represents the number of mosquitoes having completed i gonotrophic cycles at time k, and $M_i(k)$ and $U_i(k)$ represent the model predictions as described in Equations 8-13. An MCMC sampling procedure was used to obtain posterior distributions for each of the model parameters described above. These distributions were then used to calculate the DIC value for each model.

IVM Model Incorporating ATSB. In order to model the impact of ATSB in conjunction with other vector control measures, an existing ecological model of *Anopheles* dynamics [1] and an existing model of the effects of LLINs and IRS on mosquito densities [2, 3] were modified. In the combined model, mosquitoes are divided into early instars, E, late instars, L, pupae, P and unmarked and marked female adult mosquitoes, U_i and M_i, respectively, where i denotes the gonotrophic cycle number, and cycle numbers of 20 and higher are grouped into the same category. The model keeps track of mosquitoes having completed this many cycles in order to capture their distinct sporozoite rates and contributions to malaria transmission [4]. To account for larviciding with *Bacillus thuringiensis var. israeliensis* (BTI), the categories E_{BTI}, L_{BTI} and P_{BTI} are included to represent early instars, late instars and pupae, respectively, which reside in breeding sites treated with BTI. The model equations are given by,

$$\frac{dE}{dt} = (1-c)\beta_{com}(U+M) - \mu_E\left(1+\frac{E+L}{(1-c)K}\right)E - \frac{E}{d_E} \ , \quad \text{(S14)}$$

$$\frac{dE_{BTI}}{dt} = c\beta_{com}(U+M) - \upsilon\mu_E\left(1+\frac{E_{BTI}+L_{BTI}}{cK}\right)E_{BTI} - \frac{E_{BTI}}{d_E} \ , \quad \text{(S15)}$$

$$\frac{dL}{dt} = \frac{E}{d_E} - \mu_L\left(1+\gamma\frac{E+L}{(1-c)K}\right)L - \frac{L}{d_L} \ , \quad \text{(S16)}$$

$$\frac{dL_{BTI}}{dt} = \frac{E_{BTI}}{d_E} - \upsilon\mu_L\left(1+\gamma\frac{E_{BTI}+L_{BTI}}{cK}\right)L_{BTI} - \frac{L_{BTI}}{d_L} \ , \quad \text{(S17)}$$

$$\frac{dP}{dt} = \frac{L}{d_L} - \mu_P P - \frac{P}{d_P} \ , \quad \text{(S18)}$$

$$\frac{dP_{BTI}}{dt} = \frac{L_{BTI}}{d_L} - \upsilon\mu_P P_{BTI} - \frac{P_{BTI}}{d_P} \ , \quad \text{(S19)}$$

$$\frac{dU_0}{dt} = \frac{1}{2}\frac{P}{d_P} - (\mu_{M,com} + s_{0,t} + \delta_{com})U_0 \ , \quad \text{(S20)}$$

$$\frac{dM_0}{dt} = s_0 U_0 - (\mu_{M,ATSB} + \delta_{com})M_0 \ . \quad \text{(S21)}$$

The parameters in these equations and their estimates are included in Tables S3 and S4. The ATSB portion of the model allows for differences in sugar-feeding rate with gonotrophic cycle number and therefore consists of multiple differential equations. For $1 \le i \le 19$, the equations are,

$$\frac{dU_i}{dt} = \delta_{com}U_{i-1} - (\mu_{M,com} + s_{i,t} + \delta_{com})U_i \ , \quad \text{(S22)}$$

$$\frac{dM_i}{dt} = \delta_{com}M_{i-1} + s_{i,t}U_i - (\mu_{M,ATSB} + \delta_{com})M_i \ . \quad \text{(S23)}$$

And for females having completed 20 or more gonotrophic cycles, the equations are,

$$\frac{dU_{20}}{dt} = \delta_{com} U_{19} - (\mu_{M,com} + s_{20,t}) U_{20} \ , \quad \text{(S24)}$$

$$\frac{dM_{20}}{dt} = \delta_{com} M_{19} + s_{20,t} U_{20} - \mu_{M,ATSB} M_{20} \ . \quad \text{(S25)}$$

Here, the total numbers of unmarked and marked female mosquitoes are given by,

$$U = \sum_{i=0}^{20} U_i \ , \quad \text{(S26)}$$

$$M = \sum_{i=0}^{20} M_i \ . \quad \text{(S27)}$$

Initial conditions for these equations, given an equilibrium adult female population size of U_{eq}, can be calculated in the absence of interventions since these are applied after $t = 0$,

$$E(0) = 2\omega\mu_M d_L (1 + \mu_P d_P) U_{eq} \ , \quad \text{(S28)}$$

$$L(0) = 2\mu_M d_L (1 + \mu_P d_P) U_{eq} \ , \quad \text{(S29)}$$

$$P(0) = 2\mu_M d_P U_{eq} \ , \quad \text{(S30)}$$

$$E_{BTI}(0), L_{BTI}(0), P_{BTI}(0) = 0 \ , \quad \text{(S31)}$$

$$U_i(0) = \begin{cases} U_{eq}(1 - e^{-\mu_M/\delta}) e^{-i\mu_M/\delta}, i \leq 19 \\ U_{eq} e^{-i\mu_M/\delta}, i = 20 \end{cases} \ , \quad \text{(S32)}$$

$$M_i(0) = 0, \forall i \ . \quad \text{(S33)}$$

Here, ω is a function of the terms described earlier,

$$\omega = \frac{-\beta_\omega}{2} + \sqrt{\frac{\beta_\omega^2}{4} + \frac{\gamma\beta\mu_L d_E}{2\mu_E \mu_M d_L (1 + \mu_P d_P)}} \ , \quad \text{(S34)}$$

and β_ω is given by,

$$\beta_\omega = \gamma \frac{\mu_L}{\mu_E} - \frac{d_E}{d_L} + (\gamma - 1)\mu_L d_E \ . \quad \text{(S35)}$$

The environmental carrying-capacity can also be calculated as,

$$K = \frac{2U_{eq} \mu_M d_L (1 + \mu_P d_P) \gamma (\omega + 1)}{\left(\frac{\omega}{\mu_L d_E}\right) - \left(\frac{1}{\mu_L d_L}\right) - 1} \ . \quad \text{(S36)}$$

The sugar-feeding rate is given by,

$$s_{i,t} = \begin{cases} 0, t < t_{ATSB} \\ s_0, i \in \{0,1,2\}, t \geq t_{ATSB} \\ ms_0, i \geq 3, t \geq t_{ATSB} \end{cases} . \quad \text{(S37)}$$

Here, t_{ATSB} is the time at which the ATSB intervention is applied, s_0 is the rate at which female mosquitoes having completed two or less gonotrophic cycles feed on ATSB-sprayed vegetation after it has been applied, and ms_0 is the equivalent rate for female mosquitoes having completed three or more gonotrophic cycles.

LLIN and IRS coverage feeds into this model by increasing the adult mosquito death rate, $\mu_{M,com}$, and by causing female mosquitoes to take longer to find a blood-meal, thereby increasing the gonotrophic cycle length, $1/\delta_{com}$. The time at which LLINs are implemented is denoted by t_{LLIN}, and the time at which IRS is applied by t_{IRS}. Furthermore, LLIN coverage is denoted by χ_{LLIN}, which we interpret as the proportion of people reported to be sleeping under bed nets, and IRS coverage by χ_{IRS}, which we interpret as the proportion of houses sprayed with insecticide.

The effect that LLINs and IRS have on the length of the gonotrophic cycle can be divided into the time spent foraging for a blood-meal, τ_1, and the time spent resting and ovipositing by a blood-fed mosquito, τ_2. LLIN and IRS coverage has the effect of increasing the time spent foraging as a function of the probability, z, of a mosquito being repelled from an LLIN or IRS-treated house, i.e.

$$\tau_1(\chi_{LLIN}, \chi_{IRS}) = \tau_1(0,0) + z\tau_1(\chi_{LLIN}, \chi_{IRS}) = \frac{\tau_1(0,0)}{1-z} . \quad \text{(S38)}$$

Therefore, the length of the gonotrophic cycle becomes,

$$\frac{1}{\delta_{com}} = \frac{\tau_1(0,0)}{1-z} + \tau_2 . \quad \text{(S39)}$$

Following the reasoning of Le Menach *et al.* [2] and Griffin *et al.* [3], the probability of a mosquito being repelled and beginning a new search for a blood-meal is given by,

$$z = Q_0 c_{LLIN} \theta_B r_{LLIN} + Q_0 c_{IRS} \theta_I r_{IRS} + Q_0 c_{com} (\theta_I - \theta_B) r_{IRS} + Q_0 c_{com} \theta_B r_{com} , \quad \text{(S40)}$$

where,

$$c_{LLIN} = \chi_{LLIN} - \chi_{LLIN} \chi_{IRS} , \quad \text{(S41)}$$

$$c_{IRS} = \chi_{IRS} - \chi_{LLIN}\chi_{IRS} \ , \tag{S42}$$

$$c_{com} = \chi_{LLIN}\chi_{IRS} \ , \tag{S43}$$

$$r_{com} = r_{IRS} + (1 - r_{IRS})r_{LLIN} \ . \tag{S44}$$

Next, the effect that LLINs and IRS have on the adult mosquito death rate can be expressed as,

$$\mu_{M,com} = -\log p(\chi_{LLIN}, \chi_{IRS}) \ , \tag{S45}$$

where p represents the probability of a mosquito surviving one day and can be broken down into the probability, p_1, of the mosquito surviving the foraging stage and the probability, p_2, of the mosquito biting a human and surviving, i.e.,

$$p(\chi_{LLIN}, \chi_{IRS}) = (p_1(\chi_{LLIN}, \chi_{IRS})p_2)^{\delta_{com}} \ . \tag{S46}$$

The probability of the mosquito surviving the foraging stage is given by,

$$p_1(\chi_{LLIN}, \chi_{IRS}) = p_1(0,0)(w + zp_1(\chi_{LLIN}, \chi_{IRS})) = \frac{p_1(0,0)w}{1 - zp_1(0,0)} \ . \tag{S47}$$

Here, w is the probability that a surviving mosquito succeeds in feeding during a single attempt and can be calculated following the reasoning of Le Menach *et al.* (2) and Griffin *et al.* (3) to be,

$$\begin{aligned} w = 1 - Q_0 + Q_0c_0 + Q_0c_{LLIN}(1 - \theta_B + \theta_B s_{LLIN}) + Q_0c_{IRS}(1 - \theta_I + \theta_I s_{IRS}) \\ + Q_0c_{com}((\theta_I - \theta_B)s_{IRS} + 1 - \theta_I + \theta_B s_{com}) \end{aligned} \ , \tag{S48}$$

where,

$$c_0 = 1 - \chi_{LLIN} - \chi_{IRS} - \chi_{LLIN}\chi_{IRS} \ . \tag{S49}$$

Additionally, the probability of a mosquito surviving foraging in the absence of LLINs and IRS is equal to,

$$p_1(0,0) = e^{-\mu_M \tau_1(0,0)} \ , \tag{S50}$$

and the probability of it surviving resting and ovipositing is given by,

$$p_2 = e^{-\mu_M \tau_2} \ . \tag{S51}$$

Finally, both the mosquito death rate and gonotrophic cycle length feed into the β term describing the rate at which female mosquitoes oviposit eggs. In the absence of interventions, this term is given by,

$$\beta = \frac{\varepsilon\mu_M}{e^{\mu_M/\delta} - 1} , \tag{S52}$$

where ε is the number of viable eggs that a female mosquito lays per oviposition cycle and can be calculated by rearranging the subject of this formula. The new value, β_{com}, can then be calculated in the presence of interventions as,

$$\beta_{com} = \frac{\varepsilon\mu_{M,com}}{e^{\mu_M/\delta_{com}} - 1} . \tag{S53}$$

Several of the parameters introduced here vary between mosquito species and are listed in Table S4. By varying these parameters, the differential effects of interventions on *An. gambiae* and *An. arabiensis* vector densities can be modeled.

References

1. White MT, Griffin JT, Churcher TS, Ferguson NM, Basanez MG, Ghani AC: **Modelling the impact of vector control interventions on *Anopheles gambiae* population dynamics.** *Parasites and Vectors* 2011, **4**:153
2. Le Menach A, Takala S, McKenzie FE, Perisse A, Harris A, Flahault A, Smith DL: **An elaborated feeding cycle model for reductions in vectorial capacity of night-biting mosquitoes by insecticide-treated nets.** *Malar J* 2007, **6**:10
3. Griffin JT, Hollingsworth TD, Okell LC, Churcher TS, White M, Hinsley W, Bousema T, Drakeley CJ, Ferguson NM, Basáñez MG, Ghani AC: **Reducing *Plasmodium falciparum* malaria transmission in Africa: A model-based evaluation of intervention strategies.** *PLoS Med* 2010, **7**:e1000324
4. Killeen GF, McKenzie FE, Foy BD, Schieffelin C, Billingsley PF, Beier JC: **A simplified model for predicting malaria entomologic inoculation rates based on entomologic and parasitologic parameters relevant to control.** *Am J Trop Med Hyg* 2000, **62**:535-544
5. Service MW: **Studies on sampling larval populations of *Anopheles gambiae* complex.** *Bull WHO* 1971, **45**:169
6. Bayoh MN, Lindsay SW: **Effect of temperature on the development of the aquatic stages of *Anopheles gambiae sensu strict* and *An. arabiensis* larvae.** *Acta Trop* 2003, **109**:118-123
7. Kroeger A, Horstick O, Riedl C, Kaiser A, Becker N: **The potential for malaria control with the biological larvicide *bacillus-thuringiensis israelensis* (bti) in Peru and Ecuador.** *Acta Tropica* 1995, **60**:47-57
8. Killeen GF, Smith TA: **Exploring the contributions of bed nets, cattle, insecticides and excitorepellency to malaria control: A deterministic model of mosquito host-seeking behaviour and mortality.** *Trans R Soc Trop Med Hyg* 2007, **101**:867-880
9. Craig MH, Snow RW, le Sueur D: A climate-based distribution model of malaria transmission in sub-Saharan Africa. *Parasitol Today* 1999, **15**:105-111
10. Smith DL, McKenzie FE: **Statics and dynamics of malaria infection in Anopheles mosquitoes.** *Malar J* 2004, **3**:13

11. Smith DL, Dushoff J, Snow RW, Hay SI: **The entomological inoculation rate and *Plasmodium falciparum* infection in African children.** *Nature* 2005, **438**:492-495
12. Tirados I, Costantini C, Gibson G, Torr SJ: **Blood-feeding behaviour of the malarial mosquito *Anopheles arabiensis*: implications for vector control.** *Med Vet Entomol* 2006, **20**:425-437
13. Githeko AK, Service MW, Mbogo CM, Atieli FK: **Resting behaviour, ecology and genetics of malaria vectors in large scale agricultural areas of Western Kenya.** *Parassitologia* 1996, **38**:481-489
14. Killeen GF, Kihonda J, Lyimo E, Oketch FR, Kotas ME, Mathenge E, Schellenberg JA, Lengeler C, Smith TA, Drakeley CJ: **Quantifying behavioural interactions between humans and mosquitoes: evaluating the protective efficacy of insecticidal nets against malaria transmission in rural Tanzania.** *BMC Infect Dis* 2006, **6**:161
15. Mathenge EM, Gimnig JE, Kolczak M, Ombok M, Irungu LW, Hawley WA: **Effect of permethrin-impregnated nets on exiting behavior, blood-feeding success, and time of feeding of malaria mosquitoes (Diptera: Culicidae) in western Kenya.** *J Med Entomol* 2001, **38**:531-536
16. Smith A, Webley DJ: **A verandah-trap hut for studying the house-frequenting habits of mosquitoes and for assessing insecticides. 3. The effect of DDT on behavior and mortality.** *Bull Entomol Res* 1969, **59**:33-46
17. Garrett-Jones C, Shidrawi GR: **Malaria vectorial capacity of a population of *Anopheles gambiae*.** *Bull WHO* 1969, **40**:531-545
18. Charlwood JD, Smith T, Billingsley PF, Takken W, Lyimo EOL, Meuwissen JHET: **Survival and infection probabilities of anthopophagic anophelines from an area of high prevalence of *Plasmodium falciparum* in humans.** *Bull Entomol Res* 1997, **87**:445-453
