# Supplementary material for: Quantifying the mosquito’s sweet tooth: modelling the effectiveness of attractive toxic sugar baits (ATSB) for malaria vector control
Source: Malar J. 2013 Aug 23;12:291. doi: 10.1186/1475-2875-12-291 (PMC3765557; doi:10.1186/1475-2875-12-291)
Supplement: Additional file 2: Table S1 — Model comparison for basic models. [file 1475-2875-12-291-S2.doc]

**Table S1 – Model comparison for basic models**

| Model | Female ASB-feeding rate (experiment) per day, *s_f_*,*_E_* | Female ATSB-feeding rate (control) per day, *s_f_*,*_C_* | Female ATSB death rate per day, $\mu_{f,ATSB}$ | DIC* |
| --- | --- | --- | --- | --- |
| Different sugar-feeding rates, no dye decay, constant emergence | 0.50 | 0.15 | 11.7 | 779.1 |
| Different sugar-feeding rates, no dye decay, feedback emergence | 0.07 | 0.15 | 2.2 | 785.4 |
| Same sugar-feeding rates, no dye decay, constant emergence | 0.17 | 0.17 | 5.1 | 785.0 |
| Same sugar-feeding rates, no dye decay, feedback emergence | 0.11 | 0.11 | 2.6 | 808.2 |
| Same sugar-feeding rates, dye decay, constant emergence | 0.44 | 0.44 | 10.7 | 787.7 |
| Same sugar-feeding rates, dye decay, feedback emergence | 0.11 | 0.11 | 2.7 | 808.7 |

*****The best-fitting model is the one having the smallest deviance information criterion (DIC).
