# Supplementary material for: Quantifying the mosquito’s sweet tooth: modelling the effectiveness of attractive toxic sugar baits (ATSB) for malaria vector control
Source: Malar J. 2013 Aug 23;12:291. doi: 10.1186/1475-2875-12-291 (PMC3765557; doi:10.1186/1475-2875-12-291)
Supplement: Additional file 3: Table S2 — Model comparison for models incorporating gonotrophic cycle number. [file 1475-2875-12-291-S3.doc]

**Table S2 – Model comparison for models incorporating gonotrophic cycle number**

| Model | Baseline sugar-feeding rate (experiment) per day, *s*_0_,*_E_* | Baseline sugar-feeding rate (control) per day, *s*_0_,*_C_* | Sugar-feeding parameter #2 | Female ATSB death rate per day, | DIC* |
| --- | --- | --- | --- | --- | --- |
| One-step | 0.84 | 0.25 | 0.14 | 12.2 | 968.5 |
| Linear | 0.76 | 0.22 | -0.11 | 13.0 | 970.8 |
| Multiplicative | 2.89 | 0.72 | 0.24 | 11.0 | 971.7 |
| Constant | 0.45 | 0.15 | N/A | 11.0 | 979.6 |

*****The best-fitting model is the one having the smallest deviance information criterion (DIC).
