# Supplementary figures and images for: Quantifying the mosquito’s sweet tooth: modelling the effectiveness of attractive toxic sugar baits (ATSB) for malaria vector control
Source: Malar J. 2013 Aug 23;12:291. doi: 10.1186/1475-2875-12-291 (PMC3765557; doi:10.1186/1475-2875-12-291)

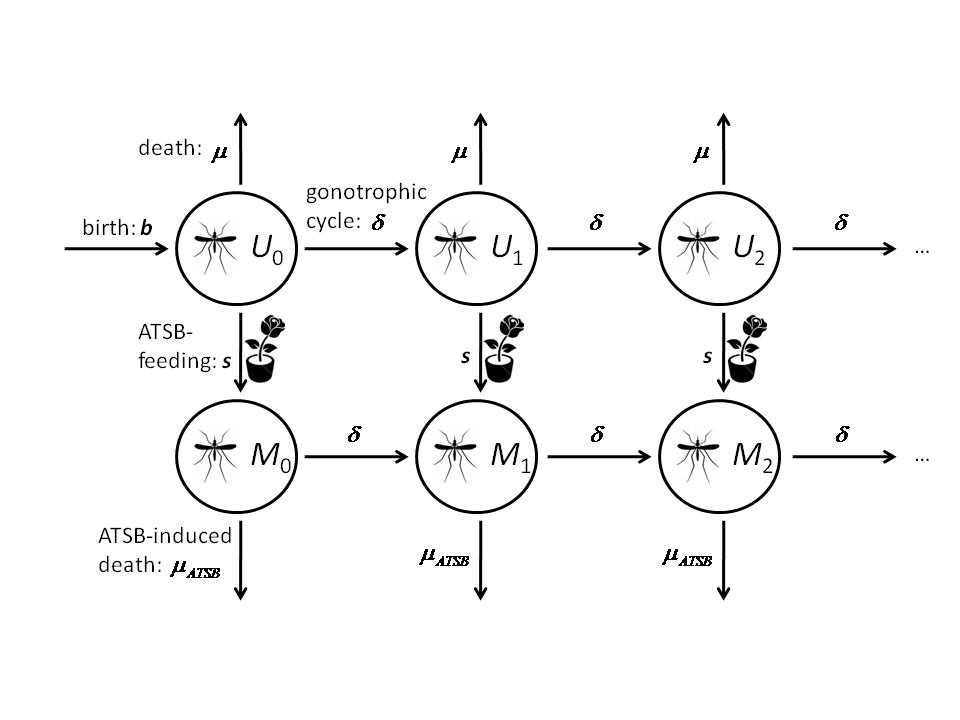

Supplement: Additional file 4: Figure S1 — Schematic of sugar-feeding model incorporating gonotrophic cycles in the experimental setting. Umarked, Ui, and marked, Mi, females are partitioned into those having completed i gonotrophic cycles, where iϵ{0,1,…,8} (mosquitoes having completed eight or more cycles are grouped into the same category). Female mosquitoes emerge at rate b into unmarked class, U0, and become marked, M0, when feeding on ATSB-sprayed vegetation at rate si. In the control setting, marked and unmarked mosquitoes die at the same rate, μ, while in the experimental setting, marked mosquitoes die at a faster rate due to the effects of the toxin, μATSB. Both marked and unmarked mosquitoes have a gonotrophic cycle length of 1/δ. [file 1475-2875-12-291-S4.tiff]

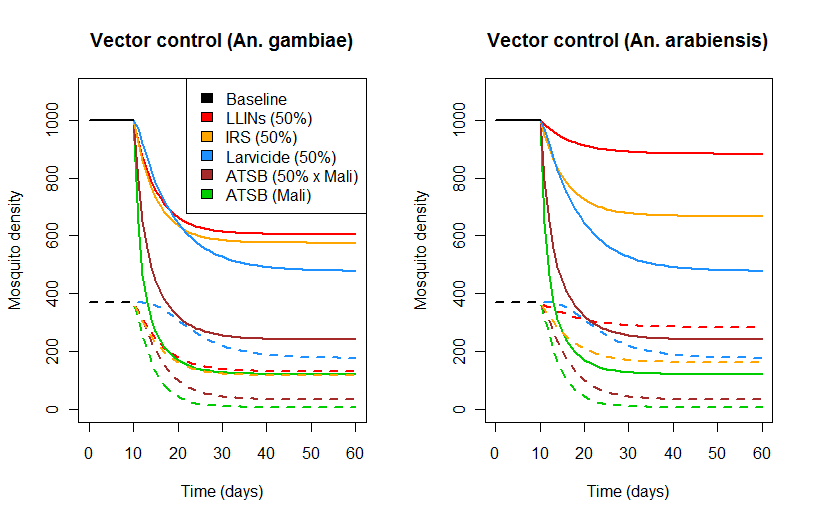

Supplement: Additional file 7: Figure S2 — Time-series depicting the effects of vector control strategies on vector density in isolation. Solid lines represent total female mosquito density, dashed lines represent females having completed three or more gonotrophic cycles. Coverage levels are assumed to be 50% for all interventions (i.e. 50% of people sleeping under nets, 50% of houses sprayed with insecticide, and 50% of breeding sites treated with BTI). ATSB is assumed to be implemented at analogous levels to that in the Mali experimental setting, and at levels such that the exposure rate would be half that of the Mali experimental setting. [file 1475-2875-12-291-S7.tiff]

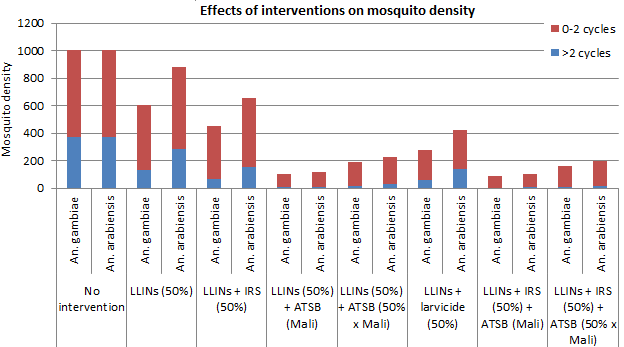

Supplement: Additional file 8: Figure S3 — Expected impact of IVM strategies on mosquito density. Red bars represent females having completed less than three gonotrophic cycles and blue bars represent females having completed three or more gonotrophic cycles. Coverage levels are assumed to be 50% for all interventions (i.e. 50% of people sleeping under nets, 50% of houses sprayed with insecticide, and 50% of breeding sites treated with BTI). ATSB is assumed to be implemented at analogous levels to that in the Mali experimental setting, and at levels such that the exposure rate would be half that of the Mali experimental setting. [file 1475-2875-12-291-S8.tiff]

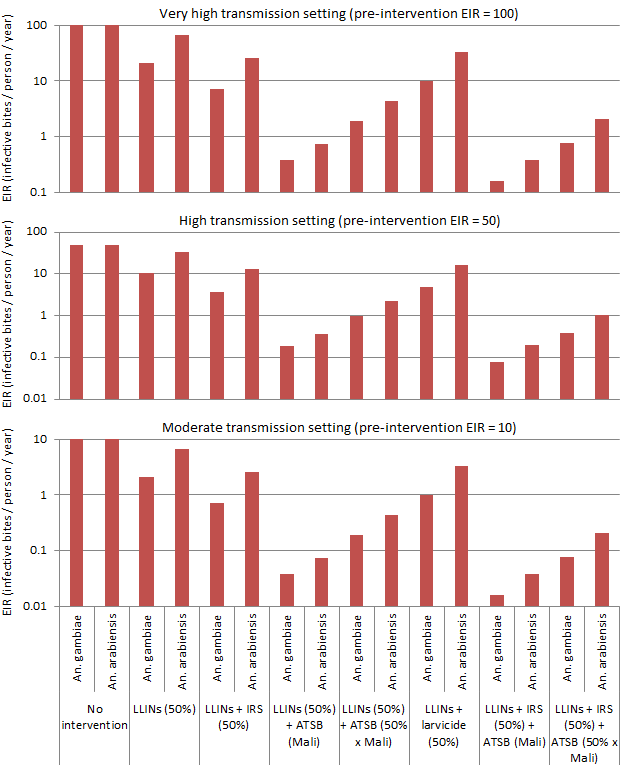

Supplement: Additional file 9: Figure S4 — Expected impact of IVM strategies on EIR. Coverage levels are assumed to be 50% for all interventions (i.e. 50% of people sleeping under nets, 50% of houses sprayed with insecticide, and 50% of breeding sites treated with BTI). ATSB is assumed to be implemented at analogous levels to that in the Mali experimental setting, and at levels such that the exposure rate would be half that of the Mali experimental setting. Model predictions are shown for three transmission settings with pre-intervention EIRs of 100 (very high transmission), 50 (high transmission) and 10 (moderate transmission). [file 1475-2875-12-291-S9.tiff]
