# Supplementary material for: Quantifying the mosquito’s sweet tooth: modelling the effectiveness of attractive toxic sugar baits (ATSB) for malaria vector control
Source: Malar J. 2013 Aug 23;12:291. doi: 10.1186/1475-2875-12-291 (PMC3765557; doi:10.1186/1475-2875-12-291)
Supplement: Additional file 5: Table S3 — Parameter estimates for IVM model that are species-invariant. [file 1475-2875-12-291-S5.doc]

**Table S3 - Parameter estimates for IVM model that are species-invariant**

| Parameter | Definition | Estimate | Reference* |
| --- | --- | --- | --- |
| $\beta$ | Number of eggs laid per adult female mosquito per day | 21.19 | [5] |
| *d_E_* | Developmental period of early instars | 6.64 days | [6] |
| $\mu_{E}$ | Death rate of early instars | 0.034 per day | [6] |
| *d_L_* | Developmental period of late instars | 3.72 days | [6] |
| $\mu_{L}$ | Death rate of late instars | 0.035 per day | [6] |
| $\gamma$ | Density-dependent factor for late instars | 13.25 | [5] |
| *d_P_* | Developmental period of pupae | 0.64 days | [6] |
| $\mu_{P}$ | Death rate of pupae | 0.25 per day | [5] |
| *C* | Coverage of breeding sites with larvicide | 0.8 | This paper |
| $\upsilon$ | Increase in death rate due to larvicide | 55.2 | [7] |
| $\delta$ | Reciprocal of gonotrophic cycle length | 0.33 per day | [4] |
| $\sigma$ | Number of gonotrophic cycles for ingested parasites to become infectious sporozoites | 3 | [4] |
| $\tau_{1}$ | Time spent foraging for a blood-meal | 0.68 days | [8] |
| $\tau_{2}$ | Time spent resting and ovipositing | 2.32 days | [8] |
| $\mu_{M}$ | Death rate of adult mosquitoes | 0.10 per day | [9] |
| $\mu_{M,ATSB}$ | Death rate of adult mosquitoes following exposure to ATSB | 17.1 per day | This paper |
| *B* | Probability than an uninfected human becomes infected after being bitten by an infectious mosquito | 0.5 | [10, 11] |
| *R* | Recovery rate of humans | 0.01 per day | [10, 11] |

*Additional_file_1.pdf
