# Supplementary material for: Quantifying the mosquito’s sweet tooth: modelling the effectiveness of attractive toxic sugar baits (ATSB) for malaria vector control
Source: Malar J. 2013 Aug 23;12:291. doi: 10.1186/1475-2875-12-291 (PMC3765557; doi:10.1186/1475-2875-12-291)
Supplement: Additional file 6: Table S4 — Parameter estimates for IVM model that vary between species. [file 1475-2875-12-291-S6.doc]

**Table S4 - Parameter estimates for IVM model that vary between species**

| Parameter | Definition | *An. gambiae*: | *An. arabiensis* | Reference* |
| --- | --- | --- | --- | --- |
| *Q*_0_ | Human blood index | 0.92 | 0.71 | [8, 12] |
| $\theta_{B}$ | Proportion of bites on a person in bed | 0.89 | 0.90 | [13, 14] |
| $\theta_{I}$ | Proportion of bites on a person indoors | 0.97 | 0.96 | [13, 14] |
| *r_LLIN_* | Probability of repeating a feeding attempt due to LLINs | 0.56 | 0.48 | [15] |
| *s_LLIN_* | Probability of feeding and surviving in presence of LLINs | 0.03 | 0.39 | [15] |
| *r_IRS_* | Probability of repeating a feeding attempt due to IRS | 0.60 | 0.60 | [16] |
| $\kappa$ | Probability that a vector becomes infectious per human bite (assuming it survives long enough) | 0.018 | 0.024 | [17, 18] |

*Additional_file_1.pdf
